# Double dissociation in radial and rotational motion sensitivity

**DOI:** 10.1371/journal.pone.0246094

**Published:** 2021-01-28

**Authors:** Nestor Matthews, Leslie Welch, Elena K. Festa, Anthony A. Bruno

**Affiliations:** 1 Department of Psychology, Denison University, Granville, OH, United States of America; 2 Department of Cognitive, Linguistic & Psychological Sciences, Brown University, Providence, RI, United States of America; University of New England, AUSTRALIA

## Abstract

Neurophysiological experiments have shown that a shared region of the primate visual system registers both radial and rotational motion. Radial and rotational motion also share computational features. Despite these neural and computational similarities, prior experiments have disrupted radial, but not rotational, motion sensitivity -a single dissociation. Here we report stimulus manipulations that extend the single dissociation to a double dissociation, thereby showing further separability between radial and rotational motion sensitivity. In Exp 1 bilateral plaid stimuli with or without phase-noise either radiated or rotated before changing direction. College students reported whether the direction changed first on the left or right–a temporal order judgment (TOJ). Phase noise generated significantly larger disruptions to rotational TOJs than to radial TOJs, thereby completing the double dissociation. In Exp 2 we conceptually replicated this double dissociation by switching the task from TOJs to simultaneity judgments (SJs). Phase noise generated significantly larger disruptions to rotational SJs than to radial SJs. This disruption pattern reversed after changing the plaids’ motion from same- to opposite-initial directions. The double dissociations reported here revealed distinct dependencies for radial and rotational motion sensitivity. Radial motion sensitivity depended strongly on information about global depth. Rotational motion sensitivity depended strongly on positional information about local luminance gradients. These distinct dependencies arose downstream from the neural mechanisms that detect local linear components within radial and rotational motion. Overall, the differential impairments generated by our psychophysical experiments demonstrate independence between radial and rotational motion sensitivity, despite their neural and computational similarities.

## Introduction

Teuber [1] provided an early neuropsychological insight by describing double dissociations. A double dissociation occurs when distinct manipulations differently affect two behaviors. For example, Teuber [1] considered two eating-related behaviors: omnivorous versus herbivorous dietary habits; quinine (bitterness) acceptance thresholds. Bitemporal lobectomies in monkeys changed dietary habits from herbivorous to omnivorous without altering quinine (bitterness) acceptance thresholds [2]. Conversely, anterior insula ablations changed quinine (bitterness) acceptance thresholds without altering dietary habits [3]. These manipulation-specific outcomes provide evidence that the two eating-related behaviors rely on at least partially independent neural events.

More generally, double dissociations provide information about potentially distinct neural contributions to similar behaviors. The more similar the behaviors, the more a double dissociation refines models of the underlying neural mechanisms. As a recent example, transcranial magnetic stimulation (TMS) experiments doubly dissociated two subtly different behavioral responses to radial motion [4]. Specifically, TMS to cortical area V3A selectively disrupted sensitivity to radial motion’s focus of expansion [4]. By contrast, TMS to cortical area MST/T02 selectively disrupted sensitivity to radial motion’s direction [4]. Here, we further that work by psychophysically demonstrating a novel double dissociation between radial and rotational motion direction sensitivity. The double dissociation provides information about *independence* between these two motion sensitivities. This independence arises in neural and computational contexts that we address next.

**Fig 1:**
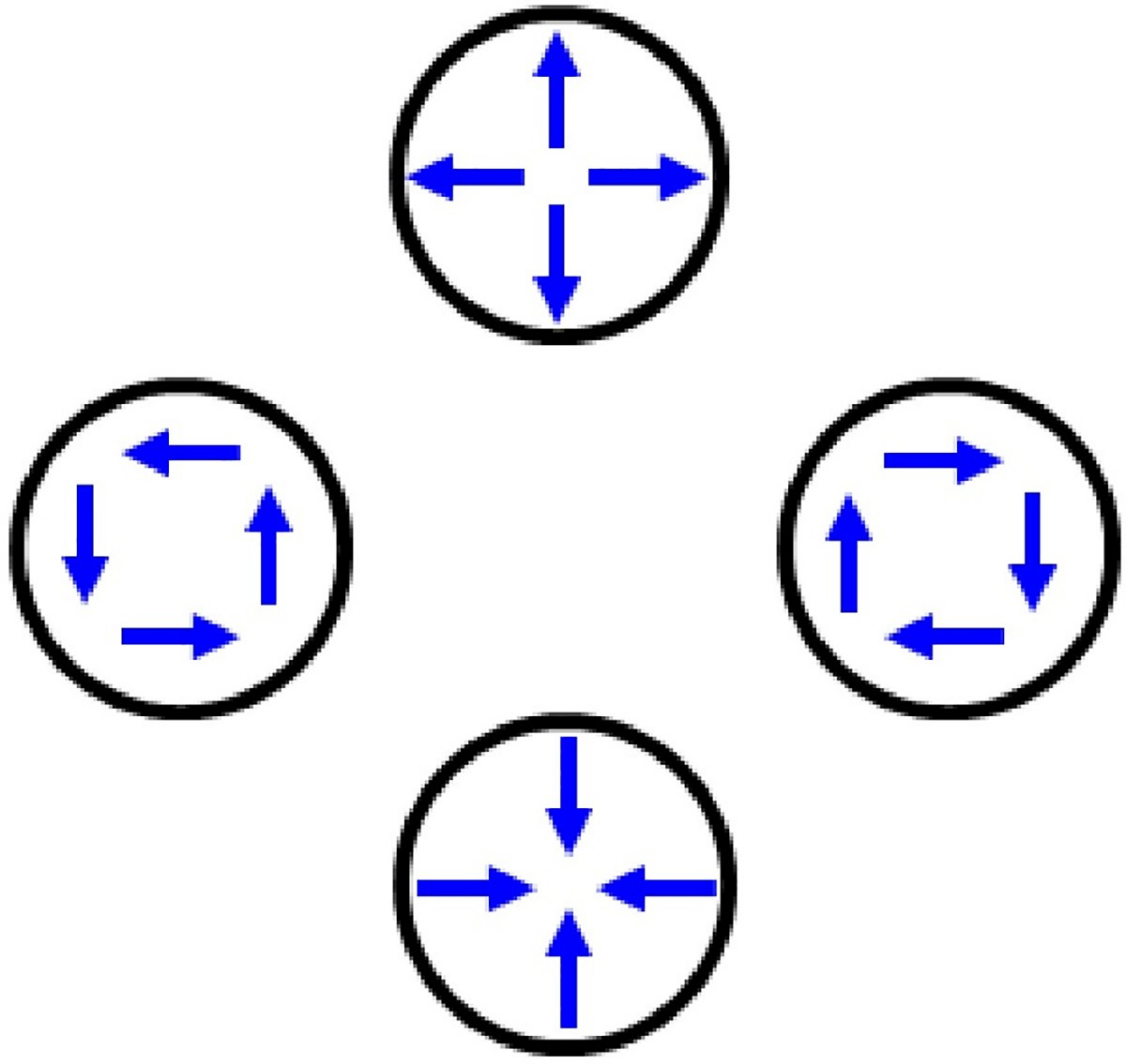
Relationships among radial, rotational, and linear motion. Rotating the local linear motion signals (arrows) 90° changes global radial motion to global rotational motion, and vice versa. Neurons in cortical area MST respond to one, two, or all three of these motion types. Modified from [19].

Neurophysiological research shows that radial and rotational motion register in the Medial Superior Temporal (MST) region of the primate visual system [5–12]. Within MST so-called “triple-component” neurons respond to three different motion types: radial, rotational, and linear motion. This conflated neural response to distinct motion types occurs despite a more parsimonious computational possibility. Specifically, neurons that register only local linear motion could parsimoniously provide radial and rotational motion sensitivity [13]. To appreciate this point, consider Fig 1. The figure's panels show the local linear components (arrows) for radial expansion (top), clockwise rotation (right), radial contraction (bottom), and anticlockwise rotation (left). Note that the global motion can change from rotational to radial—and vice versa—simply by rotating the local linear motion components 90°. Consequently, radial or rotational motion sensitivity does not require neurons tuned explicitly to radial or rotational motion. Radial and rotational motion sensitivity could emerge, in principle, from a population of local linear motion detectors alone. Interestingly, these two computational possibilities (local linear tuning, and explicit radial or rotational tuning) have well documented neurophysiological implementations. Specifically, Medial Temporal (MT) neurons that register linear motion innervate MST neurons that register rotational and radial motion [14–16]. Taken together, these neural and computational factors would tend to unify, rather than separate (dissociate) radial and rotational motion sensitivity.

**Fig 2:**
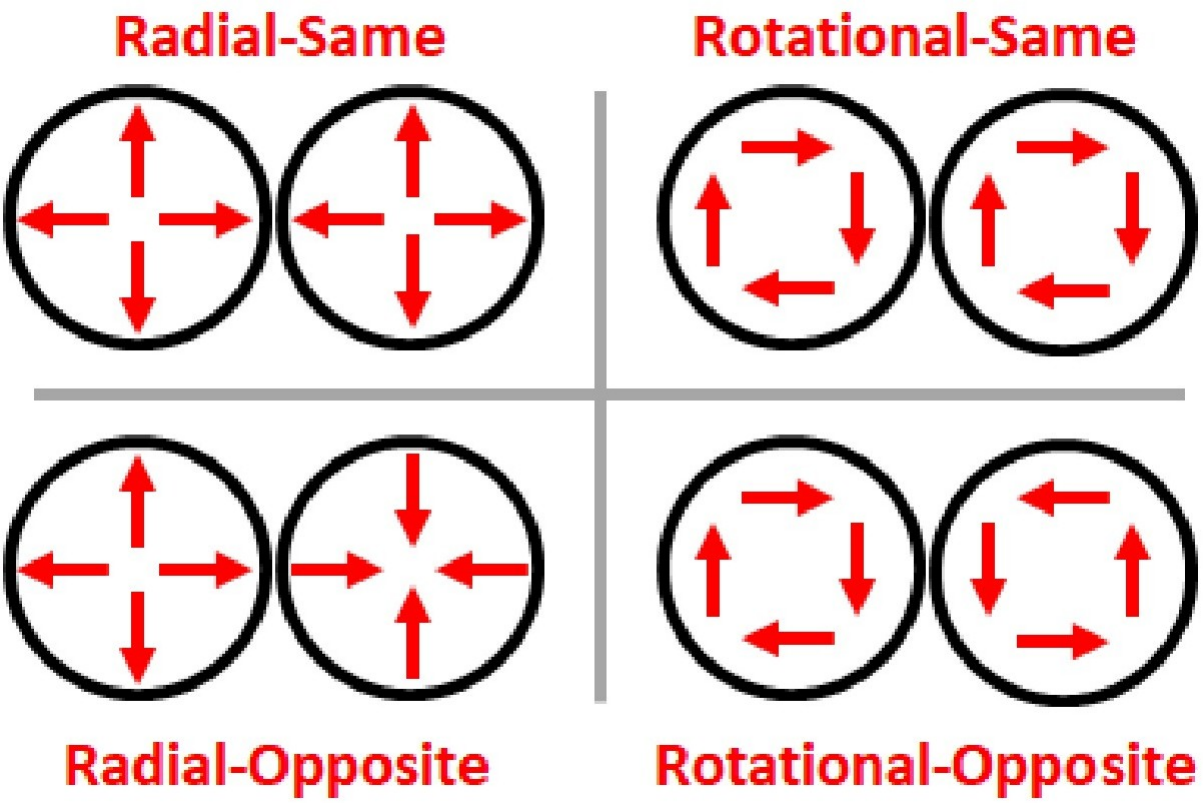
Schematic of stimuli from [19, 20]. Stimuli comprised bilaterally presented dynamic plaid pairs. On each trial both plaids radiated (left) or rotated (right), and initially moved in the same (top) or opposite (bottom) directions before reversing directions. Participants judged whether the left or right plaid changed direction first–a temporal order judgment (TOJ). Prior Radial-Opposite TOJ thresholds [19, 20] doubled or tripled relative to the other conditions, yet all conditions contained the same local linear motion components (arrows). Modified from [20].

Despite these unifying factors, diverse methodologies have converged on a single dissociation; disruptions to radial, but not rotational, motion sensitivity. For example, in a neurophysiological case study, researchers observed a stroke patient’s blindness to radial, but not rotational, motion [17]. Additional evidence comes from neurophysiological experiments that manipulated transcranial magnetic stimulation (TMS) to human cortical areas [18]. TMS to cortical area MST/T02 significantly disrupted radial motion sensitivity, whereas TMS to cortical area MT/T01 did not [18]. By contrast, rotational motion sensitivity did not exhibit this site-specific TMS effect [18]. Psychophysical manipulations have also generated the same single dissociation [19, 20]. Fig 2 schematizes the stimuli from those experiments [19, 20]. Bilaterally presented motion stimuli radiated or rotated in either the same or opposite initial directions, then reversed directions asynchronously. Participants reported whether the left or right stimulus reversed direction first -a temporal order judgment (TOJ). The change from same- to opposite-initial directions significantly impaired radial, but not rotational TOJs. Indeed, that single dissociation replicated across four populations: expert percussionists, brass players, color guard artists, and college students [19, 20].

Control experiments on the stimuli schematized in Fig 2 demonstrated that radial-TOJ specific disruptions depended on global depth perception [20]. More precisely, radial-TOJs failed because opposite-initial radial directions required participants to track counter-phased depth modulations across lateral hemifields. Intuitively, radially expanding and contracting motion provides depth information that rotational motion does not. Accordingly, here we further investigated radial and rotational motion sensitivity dissociations using a stimulus manipulation irrelevant to perceived depth. Specifically, in the present Experiment 1 we replaced the prior direction manipulation [19, 20] with a spatial phase-noise manipulation. Spatial phase noise jitters the position of the dark-light gradients within a stimulus. This manipulation presumptively introduces neural response variability in the early visual pathway mechanisms that detect moving luminance gradients. Consequently, differential effects of phase-noise on radial and rotational TOJs would point to distinct *post-*detection neural events. Critically, finding significantly greater phase-noise disruptions to rotational TOJs than to radial TOJs would complete the double dissociation. The double dissociation would provide information about the independence between these two neurally and computationally similar types of motion sensitivity.

## Experiment 1

### TOJs under a spatial-phase-noise manipulation

#### Materials and methods

The Denison University Human Participant Committee approved all experiments reported here, which we conducted with the written consent of each participant. The experiments adhere to the October 2008 Declaration of Helsinki. To promote reproducibility, the Open Science Framework [https://osf.io/knvxj/] contains the complete data set and all software necessary for replicating the experiment and the statistical analyses.

*Participants*. Fifty-five college-aged undergraduates with normal or corrected vision participated in Experiment 1. Some of the undergraduates also participated in Experiment 1 of [20], which motivated the present experiment.

*Materials and apparatus*. We used the apparatus described in [19, 20] and repeat those details here for completeness. The experiment ran on HP EliteOne 800 desktop computers, each with a Microsoft Windows 10 Enterprise operating system. Matlab 2017a software called functions from the psychophysics toolbox [21, 22]. We set the 23 inch flat screen HP LCD display’s resolution to 1920 × 1080 pixels, and the vertical refresh rate to 60 Hz. Although we did not stabilize head position, participants typically viewed the monitor from approximately 57 cm.

**Fig 3:**
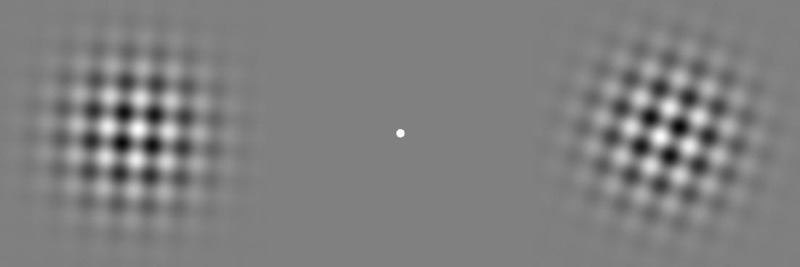
Sample movie frame. On each trial, a pair of bilaterally presented plaids either radiated or rotated. In the No-Noise condition, each plaid’s component gratings maintained a constant spatial phase across the movie’s 60 frames. In the Phase-Noise condition, the spatial phase of each plaid’s component gratings changed randomly across frames. In all conditions, the plaids within each pair moved initially in the same direction before reversing direction simultaneously or at various asynchronies. Participants indicated whether the left plaid or right plaid changed direction first—a TOJ. Modified from [19].

*Plaid stimuli*. On each trial participants viewed bilaterally presented dynamic plaid stimuli, shown in a 1-s (60-frame) movie. A sample frame from one movie appears in Fig 3. We centered each plaid 9.7° left or right of a white fixation point (152 cd/m^2^) in a gray surround (32.5 cd/m^2^). Each plaid had 95.47% Michelson contrast, a two-dimensional Gaussian window (space constant = 4.85°), and a 9.7° diameter. Within each plaid the two component gratings had identical spatial frequencies that ranged randomly across approximately three octaves (0.25–1.9 cycles/°). One of the two component orientations on each trial ranged randomly across 180°, and the other differed by 90° from that randomly selected orientation.

*Radial and rotational speeds & direction changes*. On each trial, the left and right plaid-component-gratings either shared a radial (two octaves per second) or rotational (0.102 rotations per second) speed. Those radial and rotational speeds generated comparable local linear speed gradients, which ranged between zero and 3.114° / sec. Each plaid initially moved in one radial or rotational direction for 333–666 ms, then reversed to the opposite direction for the remaining 333–666 ms. The direction reversals in the left and right plaids occurred either synchronously, or at ±67, ±133, or ±200 ms asynchronies. For illustration and ease of viewing, the Supporting Information contains sample movies (S1–S8 Movies) that display 300 ms asynchronies.

*Phase-noise manipulation*. In addition to the Motion-Type manipulation (radial, rotational) we also introduced a Phase-Noise manipulation (no-noise, phase-noise). Critically, the present no-noise condition exactly matched the “Same-Initial-Directions” condition in [19, 20]. Specifically, each plaids’ component gratings retained across frames a randomly selected *single spatial phase* (see S1–S4 Movies). Dissimilarly, across frames in the present phase-noise condition, each plaid’s component gratings *randomly varied in spatial phase over a 45° range*. Accordingly, each plaid’s luminance gradient spatially jittered throughout the 60 frames of each (1 sec) phase-noise movie (see S5–S8 Movies).

*Task & feedback*. Participants pressed either the left or right arrow key to signal whether the left or right plaid changed direction first–a TOJ. Immediate accuracy feedback followed each response. Specifically, at fixation the monitor displayed for 300 ms either the word “Correct” in green or the word “W R O N G” in red.

*Procedure*. To develop familiarity with the dynamic plaid stimuli, participants began the experiment passively viewing movies containing relatively exaggerated (300 ms) asynchronies (See Supporting Information). Participants then completed a practice block of 112 TOJ trials, separated into four block-randomized 28-trial sets. Each set comprised two Motion Types (radial, rotational) interleaved with two Phase-Noise (no-noise, phase-noise) levels and seven temporal asynchronies (0, ±67, ±133, or ±200 ms). Negative (“left lagging”) and positive (“left leading”) asynchronies corresponded respectively to trials on which the right plaid and left plaid changed direction first. When the plaids changed direction simultaneously (0 asynchrony trials) the computer pre-designated the correct response as “left first” or “right first” with equal probability.

After the 112-trial practice block, participants completed five additional 112-trial blocks (560 total trials) for analysis. These 112-trial blocks matched the practice block in all ways, including the four block-randomized 28-trial sets. Between the 112-trial blocks, each participant rested for 30 seconds while the computer displayed the participant’s cumulative percentage of correct responses. The experiment typically required about 25 minutes.

*Research design*. We administered the independent variables via a 2 x 2 (Motion-Type x Phase-Noise) within-participant experimental research design. As noted above, the computer block-randomly manipulated the within-participant independent variables: Motion-Type (radial, rotational) and Phase-Noise (no-noise, phase-noise). Control variables included counter-balancing which plaid (left, right) changed direction first, and the temporal asynchrony of the direction changes (0, ±67, ±133, or ±200 ms). We measured TOJ precision as the dependent variable.

*Statistical analyses*: *Signal detection theory*. For each participant, we used standard procedures from Signal Detection Theory [23] to evaluate time sensitivity, i.e., TOJ precision indexed by d′. Operationally, hits and false alarms occurred respectively when participants made “left first” responses and the left plaid changed direction first or second. Computationally, we determined each participant's d′ value using the formula d′ = Z_Hits_ − Z_FalseAlarms_, with the Z-distribution's SD = 0.5. Accordingly, d′ = 0.67 corresponded to non-biased 75% correct performance.

For each participant we computed four d’ values, one for each (2x2) combination of the Motion-Type and Phase-Noise variables. For each d’ value we pooled *across* negative asynchronies to determine Z_FalseAlarms_ and positive asynchronies to determine Z_Hits_. Because z-transformations require proportions greater than zero and less than one, we adopted the following procedure from [24]. For participants achieving 0 / 60 false alarms we assumed 0.5 / 60 false alarms. Conversely, for participants achieving 60/60 hits we assumed 59.5 / 60 hits. Note that pooling temporal asynchronies to estimate TOJ sensitivity (d’) parallels how a psychometric function pools temporal asynchronies to estimate TOJ thresholds.

*Inclusion / exclusion criteria*. The statistical analyses included data from each participant whose TOJ performance statistically exceeded chance (binomial test p<0.001). This inclusion criterion minimally required 57.5% correct TOJ performance across the 480 non-zero asynchrony trials for analysis. (The remaining 80 zero-asynchrony trials for analysis provided no basis for objectively determining a correct TOJ.) This resulted in excluding data from 23 participants. Data from the remaining 32 (58.2% of 55) participants appear in the Results. The 58.2% inclusion rate closely matches the 56.4% inclusion rate reported for TOJs in [20]. (We further compare inclusion rates across studies, tasks, and stimulus conditions in the General Discussion.) As noted above, the Open Science Framework [https://osf.io/knvxj/] contains the raw data from all participants.

#### Experiment 1: Results

**Fig 4:**
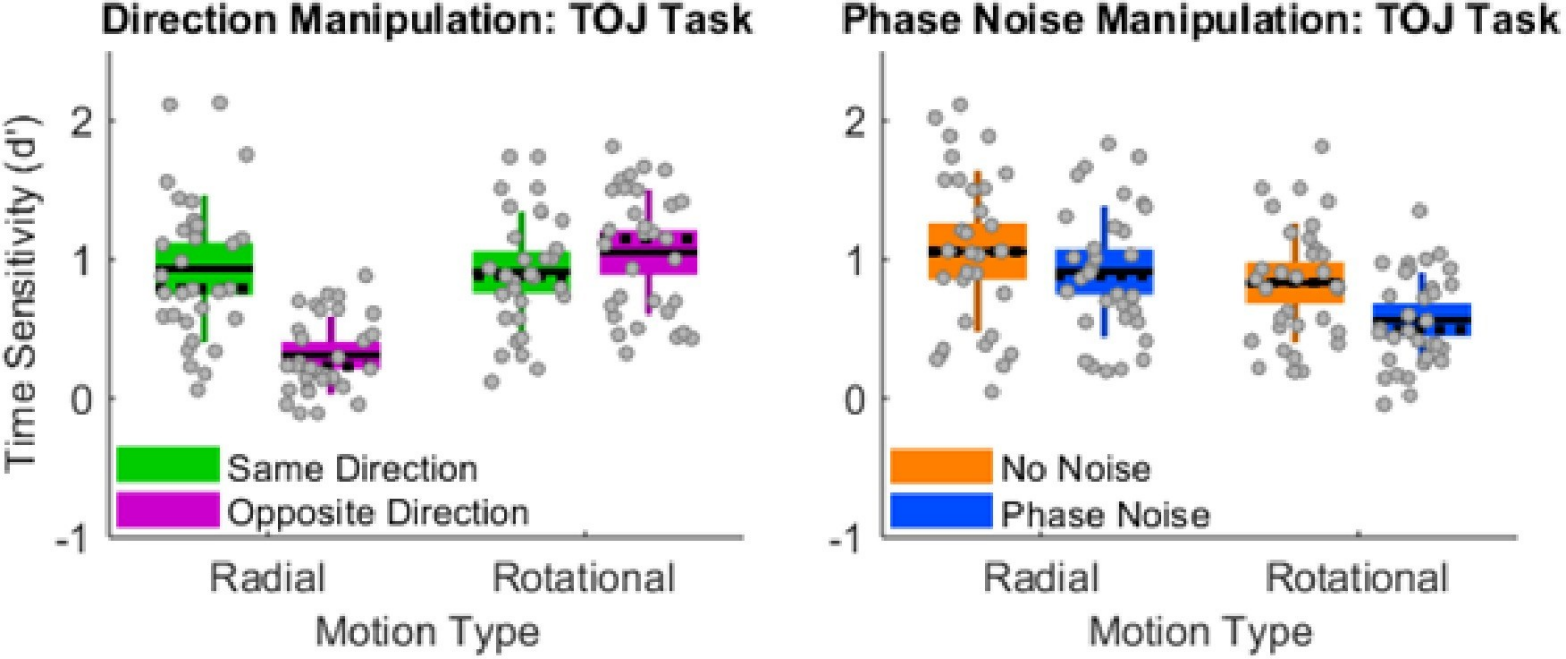
Double dissociation in radial and rotational TOJs. The prior [20] direction manipulation (left panel) demonstrated that changing from same (green boxes) to opposite (magenta boxes) initial directions impaired radial TOJs but not rotational TOJs. In the present Experiment 1 (right panel) phase noise (blue versus orange boxes) generated significantly larger impairments to rotational TOJs than to radial TOJs. Note that stimuli remained identical across the “Same Direction” (left panel, green boxes) and “No Noise” (right panel, orange boxes) conditions. The distinct 2x2 interaction patterns (left versus right panel) reveal a double dissociation between radial TOJs and rotational TOJs. Unlike standard box plots, each box extends upward and downward by 1 SD from the mean—the solid horizontal line centered within each box. The dotted horizontal line indicates the median, which overlaps with the mean in some conditions. The error bars indicate the 95% confidence interval. Gray dots correspond to individual data points, which collectively satisfied the normalcy assumption in each condition (Lilliefors test). Modified from [20].

*Double dissociation in radial and rotational TOJs*. Fig 4 juxtaposes the data from the prior [20] direction manipulation (left panel) and the present Experiment 1’s phase-noise manipulation (right panel). Visual inspection makes apparent that the prior [20] direction manipulation and present phase-noise manipulation generated distinct 2x2 patterns. These distinct 2x2 patterns arose while stimuli remained identical across the “Same Direction” (left panel, green boxes) and “No Noise” (right panel, orange boxes) conditions. Changing the initial directions from same (left panel, green boxes) to opposite (left panel, magenta boxes) markedly impaired radial TOJs and non-significantly improved rotational TOJs. The prior [20] multivariate 2x2 repeated-measures ANOVA confirmed that Motion-Type and Initial-Directions interacted significantly (F(1,30) = 67.324, p = 3.69 × 10^−9^, _p_eta^2^ = 0.692, power = 1.0). By contrast, relative to the no-noise conditions (right panel, orange boxes), phase-noise (right panel, blue boxes) impaired both radial and rotational TOJs. Moreover, visually inspecting Fig 4‘s right panel reveals that phase noise generated a greater impairment to rotational TOJs than to radial TOJs. A multivariate 2x2 repeated-measures ANOVA confirmed this significant Motion-Type x Phase-Noise interaction (F(1,31) = 4.678, p = 0.038, _p_eta^2^ = 0.131, power = 0.554). Overall, radial TOJs exhibited greater vulnerability to opposite directions while rotational TOJs exhibited greater vulnerability to phase noise. These distinct vulnerabilities reveal a double dissociation between radial and rotational TOJs.

#### Experiment 1: Discussion

Experiment 1 demonstrated that rotational TOJs exhibited significantly greater vulnerability to spatial phase noise than did radial TOJs. Conversely, prior work [19, 20] demonstrated that radial TOJs exhibited significantly greater vulnerability to opposite initial motion directions than did rotational TOJs. These complementary vulnerabilities constitute a double dissociation between radial and rotational TOJs. The double dissociation occurred despite neurophysiological and computational similarities between radial and rotational motion detection (see Introduction). Overall, the present phase-noise results complement prior evidence [4, 17–20] that radial and rotational motion sensitivity depend on separate neural events arising *after* motion detection.

These experimentally generated impairments in radial and rotational TOJs could reflect information failures at a post-motion-detection decision stage. One can model the decision stage for our TOJ task by differencing the times at which the left and right plaids changed directions.

*If [Time of Left Plaid’s Direction Change]–[Time of Right Plaid’s Direction Change] < 0*,

*then “Left First Response”*, *else “Right First Response*”.

This TOJ decision rule would perform at chance-level whenever participants lose spatial information, i.e., assigning the direction-change-times randomly to the left and right plaids. The TOJ decision rule would also perform at chance-level whenever participants lose temporal information, i.e., lacking either or both direction-change-times. These spatial and temporal information components correspond respectively to vector information (signed, positively versus negatively) and scalar information (an unsigned magnitude). Importantly, TOJs require vector information. Dissimilarly, simultaneity judgments (SJs) require only scalar information. Consequently, repeating the present and prior [20] experiments while changing the task from TOJs to SJs could distinguish vector from scalar information losses. This reasoning motivated Experiment 2.

## Experiment 2

### Conceptual replication: SJs with direction & phase noise manipulations

We conducted Experiment 2 as a *conceptual* replication of the stimulus manipulations that generated Fig 4. Unlike a direct replication, which provides evidence about test-retest reliability, a conceptual replication explores the extent to which a prior finding generalizes to new conditions. Here, the stimulus manipulations remained as before, but the *task* changed to judging whether the left and right plaids reversed direction simultaneously or not. This allowed us to exploit the distinct informational requirements of TOJs versus SJs. Similar data trends for TOJs and SJs would point toward scalar information losses, which correspond to time-based impairments here. Alternatively, finding that SJs remain robust to the stimulus manipulations that plagued TOJs (Fig 4) would point toward vector information losses, space-based impairments here.

#### Materials and methods

*Participants*. 45 college-aged Denison University undergraduates who did not participate in Experiment 1 participated in Experiment 2. All had normal or corrected vision.

*Materials and apparatus*. The materials and apparatus in Experiment 2 matched those of Experiment 1 and the prior study [20] except for the asynchronies. Specifically, in Experiment 2 the direction changes occurred either simultaneously (0 ms asynchrony) or at a 100 ms asynchrony.

*Task & feedback*. Participants pressed either the left or right arrow key to signal whether the two plaids changed direction simultaneously or asynchronously–a simultaneity judgment (SJ). Immediate accuracy feedback followed each response. Specifically, at fixation the monitor displayed for 300 ms either the word “Correct” in green or the word “W R O N G” in red.

*Procedure*. Participants began the experiment passively viewing sample movies displaying direction changes at either zero or 300 ms asynchronies. Participants then completed a practice block of 120 SJ trials that randomly interleaved the motion types (radial, rotational) and asynchronies (0, 300 ms). Left-first and right-first direction changes occurred equally often, though participants did not have to report temporal order. For 22 participants, the practice block randomly interleaved trials containing either the same or opposite initial directions–paralleling the prior study [20]. For the remaining 23 participants, the practice block randomly interleaved trials containing either phase-noise or no noise -paralleling the present Experiment 1. After the practice block all participants completed 560 trials for analysis. The trials for analysis matched the practice trials in all ways except that the direction asynchronies now occurred at either 0 or 100 ms. All other aspects of the procedure matched those of Experiment 1 and the prior study [20].

*Research design*. For the direction manipulation, we administered the independent variables via a 2 x 2 (Motion-Type x Initial-Directions) within-participant experimental research design. Likewise, for the phase-noise manipulation we administered the independent variables via a 2 x 2 (Motion-Type x Phase-Noise) within-participant experimental research design. We measured SJ precision as the dependent variable.

*Statistical analyses*: *Signal detection theory*. We modified the Signal Detection Theory [23] procedures from Experiment 1 to evaluate SJ precision, indexed by d′. Operationally, SJ hits and false alarms occurred respectively when participants classified asynchronous and simultaneous direction changes as asynchronous. For each participant in the direction manipulation we computed four d’ values. These included one for each (2x2) combination of the Motion-Type and Initial-Directions variables, or one for each (2x2) combination of the Motion-Type and Phase-Noise variables. For participants achieving 0 / 70 false alarms we assumed 0.5 / 70 false alarms. Conversely, for participants achieving 70/70 hits we assumed 69.5 / 70 hits.

*Inclusion / exclusion criteria*. The statistical analyses included data from each participant whose SJ performance statistically exceeded chance (binomial test p<0.001). This inclusion criterion minimally required 56.6% (317 / 560) correct responses across trials for analysis. This criterion resulted in excluding one participant from the initial-directions manipulation and one participant from the phase-noise manipulation. Data from the remaining 43 (95.6% of 45) participants appear in the Results. As noted above, the Open Science Framework [https://osf.io/knvxj/] contains the raw data from all participants. In the General Discussion, we consider inclusion-rate differences between Exp 2’s SJs (95.6%) and Exp 1’s TOJs (58.2%).

#### Experiment 2: Results

**Fig 5:**
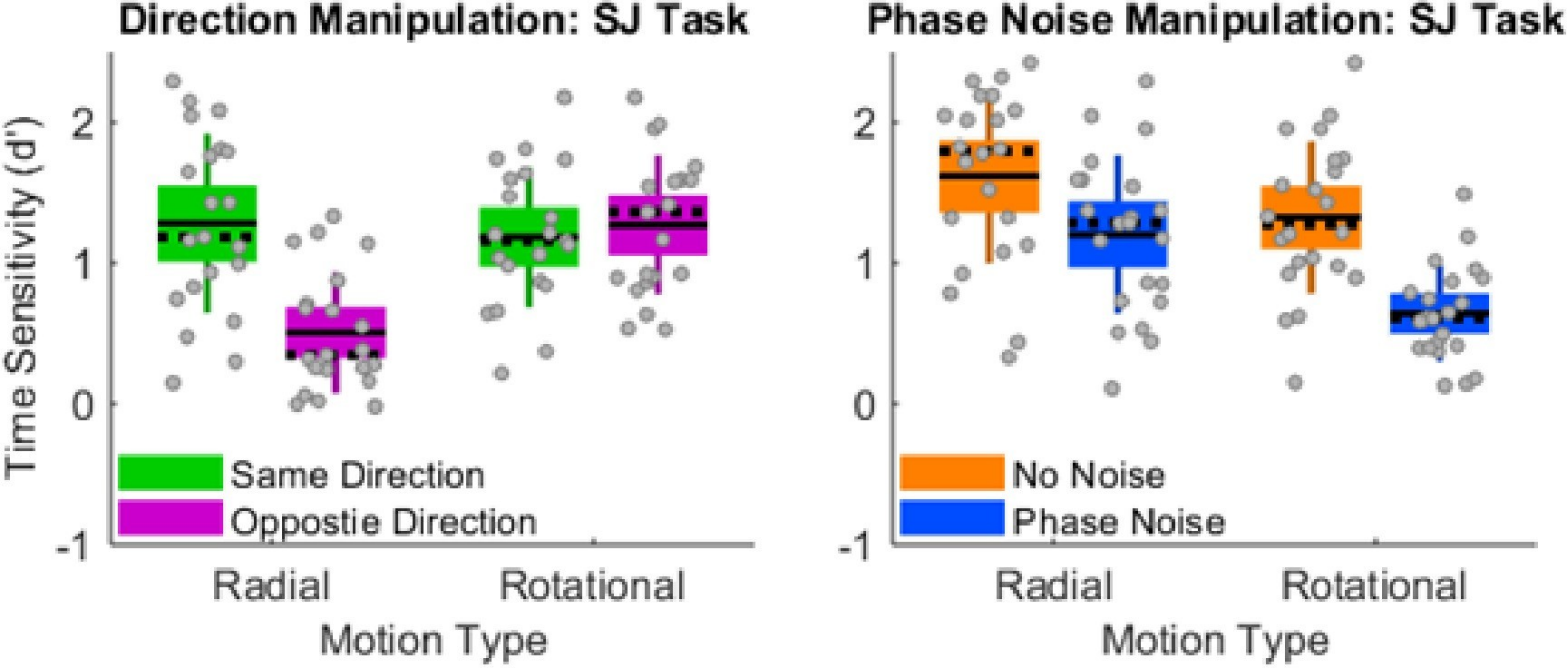
Double dissociation in radial and rotational SJs. In Experiment 2’s direction manipulation (left panel), changing from same (green boxes) to opposite (magenta boxes) initial directions impaired radial SJs but not rotational SJs. By contrast, in Experiment 2’s phase-noise manipulation (right panel), phase-noise (blue versus orange boxes) generated significantly larger impairments to rotational SJs than to radial SJs. Note that stimuli remained identical across the “Same Direction” (left panel, green boxes) and “No Noise” (right panel, orange boxes) conditions. The distinct 2x2 interaction patterns (left versus right panel) reveal a double dissociation between radial SJs and rotational SJs. Unlike standard box plots, each box extends upward and downward by 1 SD from the mean—the solid horizontal line centered within each box. The dotted horizontal line indicates the median, and the error bars indicate the 95% confidence interval. Gray dots correspond to individual data points, which collectively satisfied the normalcy assumption in each condition (Lilliefors test).

*Double dissociation in radial and rotational SJs*. Fig 5 juxtaposes the SJ data from Experiment 2’s direction manipulation (left panel) and phase-noise manipulation (right panel). Visual inspection makes apparent that the direction manipulation and phase-noise manipulation generated distinct 2x2 patterns. These distinct 2x2 patterns arose while stimuli remained identical across the “Same Direction” (left panel, green boxes) and “No Noise” (right panel, orange boxes) conditions. Changing the initial directions from same (left panel, green boxes) to opposite (left panel, magenta boxes) markedly impaired radial SJs and non-significantly improved rotational SJs. A multivariate 2x2 repeated-measures ANOVA confirmed this significant Motion-Type x Initial-Directions interaction (F(1,20) = 56.179, p = 3.14 × 10^−7^, _p_eta^2^ = 0.737, power = 1.0). A different pattern occurred in the phase-noise manipulation. Specifically, relative to the no-noise conditions (right panel, orange boxes), phase-noise (right panel, blue boxes) impaired both radial and rotational SJs. Moreover, visually inspecting Fig 5‘s right panel reveals that phase noise generated a greater impairment to rotational SJs than to radial SJs. A multivariate 2x2 repeated-measures ANOVA confirmed this significant Motion-Type x Phase-Noise interaction (F(1,21) = 9.769, p = 0.005, _p_eta^2^ = 0.318, power = 0.846).

Overall, radial SJs exhibited greater vulnerability to opposite directions while rotational SJs exhibited greater vulnerability to phase noise. These distinct vulnerabilities reveal a double dissociation between radial and rotational SJs. Importantly, the 2x2 interaction patterns for SJs in Fig 5 closely match those of TOJs in Fig 4. Experiment 2’s double dissociation in SJs therefore constitutes a conceptual replication of Experiment 1’s double dissociation in TOJs.

#### Experiment 2: Discussion

Experiment 2 revealed significant 2x2 interaction patterns in SJs that closely matched those of Experiment 1’s TOJs. The close match constitutes a conceptual replication, i.e., the data patterns generalized from TOJs to SJs when tested with the same stimulus manipulations.

The similar TOJ and SJ data patterns disconfirm vector information losses. Vector information losses in the present context correspond to spatial impairments—assigning direction-change-times randomly to the left and right plaids. Spatially random assignment would have degraded TOJs while leaving SJs intact. Instead, the significant 2x2 interaction patterns in SJs tracked with those from TOJs.

The similar TOJ and SJ data patterns match what one would predict if our stimulus manipulations generated scalar information losses. Scalar information losses in the present context correspond to temporal impairments, i.e., lacking either or both direction-change-times. A loss of either or both direction-change-times would degrade TOJs and SJs alike, consistent with our findings.

Another similarity between the TOJ data and SJ data pertains to interaction effect sizes. For TOJs and SJs alike, the direction manipulation generated substantially larger interaction effects sizes than did the phase-noise manipulation. Specifically, the direction manipulation generated interaction _p_eta^2^ values equaling 0.692 and 0.737 respectively for TOJs and SJs. By contrast, the phase-noise manipulation generated interaction _p_eta^2^ values equaling 0.131 and 0.318 respectively for TOJs and SJs. This effect-size difference arose because changing from same to opposite initial directions markedly impaired radial judgments while non-significantly improving rotational judgments. (See Figs 4 and 5.)

## General discussion

Multiple factors motivated us to investigate a double dissociation between radial and rotational motion sensitivity. First, radial and rotational motion share a computational feature. Radial motion can transform to rotational motion, and vice versa, simply by rotating local linear motion components 90°. Second, evolution appears to have exploited this computational transformation, as evidenced by the primate visual system’s innervation pattern. Specifically, Medial Temporal (MT) neurons that register linear motion innervate MST neurons that register radial and rotational motion [14–16]. Moreover, MST’s triple-component neurons conflate responses to linear, radial, and rotational motion [5, 6]. Third, despite these shared computational and neurophysiological factors, prior work identified single dissociations between radial and rotational motion sensitivity. These single dissociations occurred in the same direction; disruptions to radial motion sensitivity while leaving rotational motion sensitivity intact [17, 19, 20]. We sought to extend prior single dissociations to a double dissociation to better understand the *separability* between radial and rotational motion sensitivity.

In Experiment 1, we built upon a recently replicated direction manipulation that significantly disrupted radial but not rotational TOJs [19, 20]. Our Experiment 1’s phase noise manipulation more significantly disrupted rotational than radial TOJs, thereby completing the double dissociation. This double dissociation in radial and rotational TOJs conceptually replicated with simultaneity judgments (SJs) in Experiment 2. Here again, the direction manipulation induced greater radial than rotational disruptions; phase noise induced greater rotational than radial disruptions. Phase-noise presumptively generated neural response variability in luminance-gradient-detecting early visual pathway mechanisms during our radial and rotational trials, alike. Therefore, the significantly different disruptions that phase-noise generated implicate distinct *post-*detection neural events for radial and rotational motion sensitivity.

How might *post*-detection neural events differ for radial and rotational motion sensitivity? Our direction and phase-noise manipulations offer some information. The direction manipulation -changing the plaids’ initial directions from same to opposite- selectively disrupted radial TOJs [19, 20] and radial SJs. Notably, among the four conditions tested, the radial-opposite condition uniquely generated monocular depth cues to globally counter-phased depth modulations. Radially expanding plaids cued looming depth in one hemifield while radially contracting plaids cued receding depth in the other. TOJs [19, 20] and SJs (Experiment 2) improved significantly when the plaid motion cued single or bilaterally synchronized depth planes. This indicates that radial motion sensitivity depends strongly on depth cue information. By contrast, rotational motion sensitivity appears to depend strongly on local positional information about luminance gradients. The evidence for this comes from our phase-noise manipulation. Phase-noise positionally jittered the dark-light gradients within each plaid. This positional noise produced greater rotational than radial disruptions for TOJs and SJs, alike. Critically, that finding also disconfirms the parsimonious possibility that linear motion detectors controlled radial and rotational motion sensitivity [13, 25–31].

The radial and rotational motion double dissociations reported here align well with prior speed discrimination findings. One study [32] measured radial and rotational speed discrimination thresholds and repeated those measurements within participants on a second day. Radial speed discrimination thresholds from day 1 correlated significantly with those from day 2. Likewise, rotational speed discrimination thresholds from day 1 correlated significantly with those from day 2. These significant within-motion-type correlations established test-retest reliability and captured 32% - 65% of the speed-discrimination threshold variability. By contrast, radial and rotational speed discrimination thresholds failed to correlate significantly with each other within each daily session. Indeed, the within-session radial / rotational correlations captured only 0%– 14% of the speed discrimination threshold variability. The pattern of strong within-motion-type correlations and weak between-motion-type correlations does not, by itself, constitute a double dissociation. However, the pattern matches what one would expect if distinct neural rules governed radial and rotational motion sensitivity. This converges with the double dissociations in radial and rotational motion sensitivity reported here for TOJs and SJs.

The conceptual replication reported here for TOJs *and* SJs seems surprising, as prior studies distinguish these two tasks. For example, TOJs on dynamic dual-stream displays revealed significantly hastened perception of left visual field (LVF) targets [33]. When the same participants viewed the same stimuli but made SJs rather than TOJs, the hastened LVF perception disappeared [33]. TOJs and SJs also exhibit distinct reaction time patterns and training effects, reflecting distinct TOJ and SJ decision rules [34]. Moreover, TOJs and SJs appear to differ in difficulty, as evidenced by the inclusion rates in Table 1. Table 1‘s inclusion rates come from two prior studies [19, 20] (gray shading) and the present Experiments 1 (yellow shading) and 2 (cyan shading). The two prior studies [19, 20] measured radial and rotational motion TOJs with plaid stimuli and the temporal asynchronies used in the present Experiment 1. On TOJs, inclusion rates for percussion, brass, and color guard experts [19] notably exceeded those for college students [20, and the present Experiment 1]. Yet, among college students who viewed plaids with phase noise, inclusion rates increased markedly after merely changing the task from TOJs (58.2%) to SJs (95.6%). This suggests greater difficulty for TOJs than for SJs.

**Table 1:** Inclusion rates across radial and rotational motion studies, stimuli, and tasks.

	Percussion	Brass	Color Guard	College	College	College
	Experts	Experts	Experts	Students	Students	Students
	TOJs	TOJs	TOJs	TOJs	TOJs	SJs
	No Noise	No Noise	No Noise	No Noise	Phase Noise	Phase Noise
**Total N**	25	71	36	55	55	45
**Included**	25	67	29	31	32	43
**Excluded**	0	4	7	24	22	2
**Inclusion %**	*100%*	*94*.*4%*	*80*.*6%*	*56*.*4%*	*58*.*2%*	*95*.*6%*

The table summarizes inclusion rates from two prior radial and rotational motion studies [19, 20] (gray) and the present Experiments 1 (yellow) and 2 (cyan). The studies collectively comprised 287 college-aged participants. The task (TOJs) and stimuli (plaids with no phase noise) remained *identical* across the prior studies [19, 20] (gray). Yet, inclusion rates for expert percussionists (100%), brass players (94.4%), and color guard artists (80.6%) markedly exceeded those of age-matched college students (56.4%). College students’ inclusion rates remained comparably low when making TOJs in the absence (56.4%) or presence of stimulus phase noise (58.2%). By contrast, when college students viewed stimuli with phase noise, inclusion rates approached the ceiling after changing the task from TOJs (58.2%) to SJs (95.6%). Overall, the table demonstrates group temporal-sensitivity differences within age-matched populations, and greater difficulty for TOJs than SJs among college students.

Despite their differences, the present TOJ and SJ tasks *each* revealed a double dissociation between radial and rotational motion sensitivity. Why? A possible explanation arises from the nature of the information losses that our direction and phase-noise manipulations generated. In principle, these manipulations could have disrupted spatial (hemifield) information only. This would have forced participants to assign direction-change-times randomly to our left and right plaids. Assigning direction-change-times randomly to our left and right plaids would impair TOJs, but not SJs. The present TOJs required spatial (hemifield) information *and* temporal (direction-change-time) information. By contrast, the present SJs required only temporal (direction-change-time) information. Interestingly, the difference between SJ (95.6%) and TOJ (58.2%) participant-inclusion rates (see Table 1) might reflect this reprieve from tracking spatial information. Temporal information losses, alone, would parsimoniously explain the radial and rotational double dissociations reported here for TOJs *and* SJs.

Lastly, we conclude by speculating about the relationship between temporal information losses and global depth cues from radial motion. Radial TOJs and SJs may have suffered temporal information losses arising from difficulties in tracking globally *counter-phased* depth cues. Conversely, globally *synchronized* depth cues may have provided reliable temporal information for radial TOJs and SJs in phase noise. This would explain why our phase noise generated significantly better radial than rotational TOJs and SJs. Stated differently, radial motion generates salient depth cues that might act as a double-edged sword. Globally counter-phased depth cues (radial opposite condition) can impair the detection of temporal information (direction-change-times) [17, 19, 20]. Conversely, globally *synchronized* depth cues (radial same condition) can *facilitate* the detection of temporal information (direction-change-times). *Globally* synchronized depth cues might provide compensatory information to counteract phase-noise that corrupts *local* positional information about luminance gradients. Though speculative, disruption from globally counter-phased depth and facilitation from globally synchronized depth would concisely explain our results.

## Conclusion

We report novel double dissociations between radial and rotational motion sensitivity. These dissociations conceptually replicated across distinct tasks: temporal order judgments (TOJs) and simultaneity judgments (SJs). The double dissociations revealed distinct dependencies for radial and rotational motion sensitivity. Radial motion sensitivity depended strongly on information about global depth. Rotational motion sensitivity depended strongly on positional information about local luminance gradients. These distinct dependencies arose downstream from the neural mechanisms that detect local linear components within radial and rotational motion. Overall, the differential impairments generated by our psychophysical experiments demonstrate independence between radial and rotational motion sensitivity, despite their neural and computational similarities.

## Supporting information

S1 Movie No-noise radial stimuli, with left-first direction change. (MP4)

S2 Movie No-noise radial stimuli, with right-first direction change. (MP4)

S3 Movie No-noise rotational stimuli, with left-first direction change. (MP4)

S4 Movie No-noise rotational stimuli, with right-first direction change. (MP4)

S5 Movie Phase-noise radial stimuli, with left-first direction change. (MP4)

S6 Movie Phase-noise radial stimuli, with right-first direction change. (MP4)

S7 Movie Phase-noise rotational stimuli, with left-first direction change. (MP4)

S8 Movie Phase-noise rotational stimuli, with right-first direction change. (MP4)
